# The effect of feeding fermented distillers' grains diet on the intestinal metabolic profile of Guanling crossbred cattle

**DOI:** 10.3389/fvets.2023.1238064

**Published:** 2023-10-20

**Authors:** Xiaofen Luo, Tiantian Zhang, Duhan Xu, Mingming Zhu, Junjie Zhang, Rong Zhang, Qian Hu, Yongxuan Wang, Guangxia He, Ze Chen, Shihui Mei, Bijun Zhou, Kaigong Wang, Chao Chen, Erpeng Zhu, Zhentao Cheng

**Affiliations:** ^1^College of Animal Science, Guizhou University, Guiyang, China; ^2^Key Laboratory of Animal Diseases and Veterinary Public Health of Guizhou Province, College of Animal Science, Guizhou University, Guiyang, China

**Keywords:** fermented distiller's grains (FDG), Guanling crossbred cattle, intestinal metabolic profile, metabolites, metabolic pathways

## Abstract

Fermented distiller's grains (FDG)-based diets are nutritious and can improve the growth and intestinal immunity in livestock. However, there is limited research examining the effect of feeding FDG-based diets on changes in intestinal metabolites and related pathways in livestock. In this study, nine Guanling crossbred cattle (Guizhou Guanling Yellow cattle × Simmental cattle) were selected and randomly divided into a basal diet (BD) group and two experimental groups fed with FDG replacing 15% and 30% of the daily ration concentrates (FDG-Case A and FDG-Case B), respectively, with three cattle in each group. Fresh jejunum (J) and cecum (C) tissues were collected for metabolomic analysis. Differential metabolites and metabolic pathways were explored by means of univariate and multivariate statistical analysis. Compared with the J-BD group, 30 and 100 differential metabolites (VIP > 1, *p* < 0.05) were obtained in the J-FDG-Case A group and J-FDG-Case B group, respectively, and the J-FDG-Case B vs. J-FDG-Case A comparison revealed 63 significantly differential metabolites, which were mainly divided into superclasses including lipids and lipid-like molecules, organoheterocyclic compounds, and organic acids and derivatives. Compared with the C-BD, 3 and 26 differential metabolites (VIP > 1, *p* < 0.05) were found in the C-FDG-Case A group and C-FDG-Case B group, respectively, and the C-FDG-Case B vs. C-FDG-Case A comparison revealed 21 significantly different metabolites, which were also mainly divided into superclasses including lipids and lipid-like molecules, organoheterocyclic compounds, and organic acids and derivatives. A total of 40 metabolic pathways were identified, with a significance threshold set at *p* < 0.05. Among them, 2, 14, and 18 metabolic pathways were significantly enriched in the J-FDG-Case A vs. J-BD, J-FDG-Case B vs. J-BD, and J-FDG-Case B vs. J-FDG-Case A comparisons, respectively. Meanwhile, 1, 2, and 3 metabolic pathways were obtained in the C-FDG-Case A vs. C-BD, C-FDG-Case B vs. C-BD, and C-FDG-Case B vs. C-FDG-Case A comparisons, respectively. Furthermore, four significant metabolic pathways, namely insulin resistance, biosynthesis of unsaturated fatty acids, linoleic acid metabolism, and primary bile acid biosynthesis, were significantly enriched in Guanling crossbred cattle fed FDG diets. These results suggest that feeding FDG diets may promote the growth and intestinal immunity of Guanling crossbred cattle by regulating metabolic patterns of lipid compounds and related metabolic pathways. This study sheds light on the potential metabolic regulatory mechanisms of FDG diets and offers some references for their use in livestock feed.

## 1. Introduction

With the rapid advancement of China's agricultural industry in recent years, there has been a surge in costs for essential raw materials such as corn and bran, which in turn has resulted in elevated animal breeding expenses (1). Distillers' grains (DGs) are a plentiful, readily available, and cost-effective alternative resource in China, with a production of 20–30 million tons in 2020. DGs are worth reusing due to their high content of crude protein, fiber, fat, and vitamins, making them an excellent alternative resource for animal feed (2). The growing use of fermented distillers' grains (FDGs) has been evidenced in the livestock industry, including cattle, pigs, and broilers, which can improve the production performance and meat quality of the animals (3–5). Evidence has shown that the supplement of fermented distillers' grains (FDGs) to cattle diet can increase weight gain, improve slaughter performance, including crude protein content, and reduce the feed-to-weight ratio in cattle (6, 7). Additionally, supplementing beef cattle diets with FDGs can increase the protein content of the resulting beef (8). These studies suggest a promising potential for the utilization of FDGs in animal feeds, which promotes animal growth and development and improves beef quality. A recent study demonstrated that feeding Moutai FDG enhanced meat quality by increasing back muscle thickness, intramuscular protein content, and fat content, as well as upregulating the expression of various lipid metabolism molecules in crossbred cattle, suggesting a positive regulation of the Moutai FDG diet on slaughter performance and muscle lipid metabolism (9).

The intestine is an organ with a special tubular structure that is primarily responsible for digesting food and absorbing water, inorganic salts, and nutrients (10). Physiological and pathological events that trigger disturbances of intestinal homeostasis can negatively affect intestinal health and cause intestinal diseases that affect organism growth and development (11). The intestine also serves as the largest immune organ and the frontline for immune defense in the body, whose health is related to the occurrence of intestinal diseases and the multi-system regulation of the entire body (12, 13). The maintenance of gut balance is associated with a variety of factors, including the immune system, the microbial ecosystem, and the diet. Additionally, the gut plays an important role in the regulation of systemic physiology, metabolism, and immunity (14). Evidence has shown that fermented diets can produce short-chain fatty acids (SCFAs), phenolics, and other small metabolic molecules that not only contribute to maintaining the balance between the animal's intestinal barrier function and microbial community but also provide energy to the intestinal epithelial cells, and such substances also have antioxidant properties, which reduce oxidative stress and inflammatory responses in the intestine (15–20). To date, most of the studies mentioned above have focused on the regulation of intestinal flora structure and metabolism in intestinal contents, with little attention to intestinal (wall) metabolism. The change of indices in the intestinal contents reflects to some extent the combined action of the body and the intestinal microorganisms, while the metabolic profile of the intestinal wall is more biased toward indicating changes in the body's metabolism. In this study, we investigated the effect of feeding FDG diets on the intestinal metabolic profile in Guanling crossbred cattle via LC-MS-based non-targeted metabolomics. The results will provide new insights into the regulatory effects of dietary FDG supplementation on intestinal metabolites and their metabolic pathways in ruminants, which will be beneficial for further development and utilization of FDGs as a potentially valuable feed resource.

## 2. Materials and methods

### 2.1. Origin and treatment of FDGs

The DGs used in this study were produced by Kweichow Moutai Group in Moutai Town, Renhuai City, Guizhou Province, China. The main ingredients of the fermentation substrate include 92% fresh DG, 3% corn flour, 3% rapeseed meal, and 2% wheat bran. After the fermentation substrate was evenly mixed with a feed mixer, 50 kg of microbial starter and water mixture were added, which was prepared by mixing 100 g of biological fermentation agent with 5 kg of water containing 0.1% brown sugar. The mixture is added while stirring until it is completely added. After activation for 3–5 days, the DGs were bagged and sealed and then fermented for 9 days to obtain the FDGs (the actual cost of FDGs in either the 15% or 30% FDG group is approximately 0.4 RMB/kg) to be used for subsequent experiments. The components of the microbial starter included lactic acid bacteria, yeast, *Bacillus, Bifidobacterium, Clostridium butyricum*, amylase, protease, cellulase, and lipase.

### 2.2. Animal experiment

This study was conducted at the Yueyuan cattle farm located in Guanling County, Anshun City, Guizhou Province (105° 58′ E, 25° 98′ N, altitude, approximately 1863 m). The experimental period was from September 2020 to November 2020, during which the average minimum outdoor temperature was 16.9°C and the maximum temperature was 25.6°C. Nine Guanling crossbred cattle (Guizhou Guanling Yellow cattle × Simmental cattle) in good health and of similar age (18 months old), weighing 300 ± 25 kg, were provided by the beef cattle fattening farm of Guanling County Yellow Cattle Group. Laboratory tests showed that these cattle were negative for infection with *Brucella, Mycobacterium tuberculosis*, foot-and-mouth disease virus, and lumpy skin disease virus. These cattle were ear-marked and randomly divided into a basal diet (BD) group, a Case A group, and a Case B group (containing 0%, 15%, and 30% FDG, respectively, to replace part of the concentrates), with three cattle in each group. The composition and nutrient content of the diets are shown in Supplementary Table S1 (21). The diets were thoroughly mixed to form a complete mixed diet and fed twice daily (9:00 a.m. and 4:30 p.m.). Cattle in each group were housed in separate pens and fed free water under identical housing conditions throughout the duration of the experiment.

### 2.3. Collection and processing of samples

On the 60th day of the formal feeding period, the animals underwent overnight fasting, and then tissues were collected from the jejunum, namely jejunum-BD (J-BD), jejunum-FDG-Case A (J-FDG-Case A), and jejunum-FDG-Case B (J-FDG-Case B), and from the cecum, namely cecum-BD (C-BD), cecum-FDG-Case A (C-FDG-Case A), and cecum-FDG-Case B (C-FDG-Case B). The collected tissues were then rinsed with RNase-free saline. QC samples were obtained by mixing equal volumes of the extracts from all collected samples. Prior to the metabolomics analysis, 30 mg of intestinal tissue samples were added to a 1.5-mL tube with 20 μL of L-2-chlorophenylalanine (0.3 mg/mL) dissolved in methanol as internal standard, and then 400 μL mixture of methanol/water (1/4, v/v) was added and pre-chilled at −20°C for 2 min and then ground in a grinder for 2 min. All the samples were subjected to ultrasonic extraction for 10 min, stored at −20°C for 30 min, and then centrifuged at 13,000 × *g* for 10 min at 4°C. A total of 300 μL of supernatants were dried in a freeze-concentration centrifugal dryer and resolubilized by 200 μL of methanol/water (1/4), vortexed for 30 s, and subjected to ultrasonic extraction for 3 min. After placement for 2 h at −20°C, the samples were centrifuged at 4°C (13,000 × *g*) for 10 min, and 150 μL of supernatants were filtered through 0.22-μm microfilters and then transferred to liquid chromatography (LC) vials (22).

### 2.4. LC-MS analysis

The LC-MS analysis of intestinal samples in both ESI positive and ESI negative ion modes was conducted using an ACQUITY UPLC I Class system (Waters Corporation Milford, USA), coupled with a VION IMS Q-TOF mass spectrometer (Waters Corporation Milford, USA). A 1-μL aliquot of intestinal tissue samples was injected into an ACQUITY UPLC™ BEH C18 column (50 × 2.1 mm i.d., 1.7 μm; Waters Corporation, Milford, MA, USA). Mobile phase A consisted of 0.1% formic acid in water, and mobile phase B comprised a mixture of acetonitrile/methanol (2/3) containing 0.1% formic acid (v/v). The flow rate was 0.4 mL/min. The conditions for UPLC separation and ESI-VIONIMS Q-TOF detection are shown in Supplementary Tables S2, S3. The QC samples were utilized to evaluate the reproducibility and reliability of the LC-MS system, which contains metabolic information that enables the assessment of the stability of the mass spectrometry system. The ion peaks with a relative standard deviation of >0.4 for the QC group samples were removed. The stability of the system was then assessed using principal component analysis (PCA). The PCA model plot was obtained after seven cycles of cross-validation to verify whether the QC samples were closely clustered together, thus judging the stability of the instrument's detection.

### 2.5. Data analysis

For metabolomics data processing, the obtained LC-MS raw data were initially analyzed using the Progenesis QI v2.3 software (Non-linear Dynamics, Newcastle, UK). The metabolites were qualitatively analyzed by utilizing the Human Metabolome Database (HMDB), Lipid Maps (v2.3), the METLIN database, and a self-developed database. For the extracted data, ion peaks with missing values (0 value)>50% were eliminated, the 0 value with half of the minimum value was replaced, and the qualitatively obtained compounds were screened according to the qualitative result evaluation (score). The qualitative results were screened between 36 and 60 points, below which they were considered inaccurate and deleted. Finally, the positive and negative ion data were combined into a data matrix containing all the information extracted from the original data necessary for analysis. Subsequently, multivariate statistical analyses were carried out using SIMCA 14.0 (Umetrics AB, Umeå, Sweden).

The PCA and orthogonal partial least squares discriminant analysis (OPLS-DA) were performed to reveal the global metabolic changes between the BD and FDG groups using SIMCA 14.0. An unsupervised PCA was first used to examine both the metabolic differences between the groups and the individual metabolic differences within samples. To maximize the differences within the model, a supervised OPLS-DA was performed on metabolites between groups. In addition, to prevent overfitting of the model, the quality of the model was examined by response permutation testing with 200 responses. Then, univariate analyses were performed on the sample data, which consisted of a *t-*test and fold change analysis. The fold change (FC) value was calculated through a fold analysis of variance to assess differences in metabolite expression between the two groups. The *p-*values were obtained using a *t*-test, and then volcano plotting was performed using Origin 2020 (Origin Lab, Northampton, MA, USA) to visualize the *p-*values and FC to facilitate the rapid identification of metabolites with significant differences. Multivariate and univariate statistical analyses were combined to screen the differential metabolites between groups.

### 2.6. Key biomarkers and metabolic pathway analysis

To reveal the mechanism of metabolic pathway variation in intestinal samples, an analysis of differential metabolites was carried out with metabolic pathway enrichment analysis based on the Kyoto Encyclopedia of Genes and Genomes (KEGG) database (http://www.kegg.jp/kegg/pathway.html). Their KEGG IDs and pathways were obtained, and then the number of metabolites enriched in each corresponding pathway was calculated. The pathway with a *p* < 0.05 was selected as an enriched pathway. The formula for calculating *p*-values is provided as follows:


$$\begin{array}{l} P=1-\overset{m-1}{\underset{i=0}{\sum }}\frac{\left(\begin{array}{c} M\\ i \end{array}\right)-\left(\begin{array}{c} N-M\\ n-i \end{array}\right)}{\left(\begin{array}{c} N\\ n \end{array}\right)} \end{array}$$

where N: the total number of metabolites, n: the number of differential metabolites, M: the number of metabolites annotated as a specific pathway, and m: the number of differential metabolites annotated as a specific pathway. A lower *p-*value indicates a more significant difference within the metabolic pathway.

### 2.7. Statistical analyses

All continuous variables were expressed as the mean ± standard deviation (M ± SD). The differences between the groups were analyzed using Student's *t*-test, where the significant difference was set at a *p* < 0.05. All statistical analyses were performed using SPSS Statistics 26.0 (Chicago, IL, USA).

## 3. Results

### 3.1. Quality control of the metabolomics of jejunum and cecum tissues

**Figure 1:**
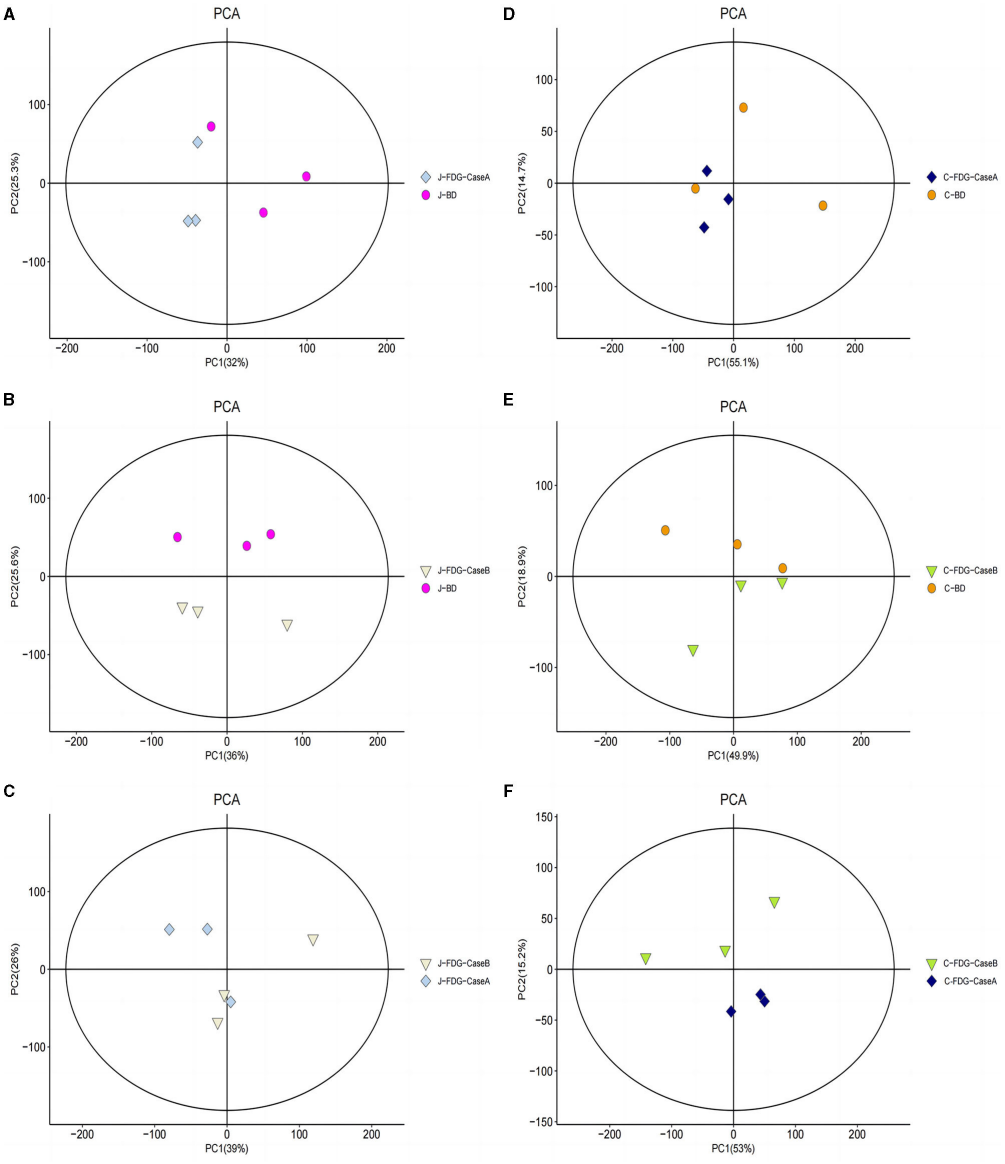
PCA model score of the untargeted metabolomics approach. **(A)** J-FDG-Case A vs. J-BD comparison (R^2^X = 0.786), **(B)** J-FDG-Case B vs. J-BD comparison (R^2^X = 0.781), **(C)** J-FDG-Case B vs. J-FDG-Case A comparison (R^2^X = 0.810), **(D)** C-FDG-Case A vs. C-BD comparison (R^2^X = 0.828), **(E)** C-FDG-Case B vs. C-BD comparison (R^2^X = 0.835), and **(F)** C-FDG-B vs. C-FDG-Case A comparison (R^2^X = 0.818; *p* < 0.05). Each dot, triangle, or diamond on the plot represents a sample in the corresponding group. BD, basal diet group; FDG-Case A and FDG-Case B: 15% FDG group and 30% FDG group.

**Figure 2:**
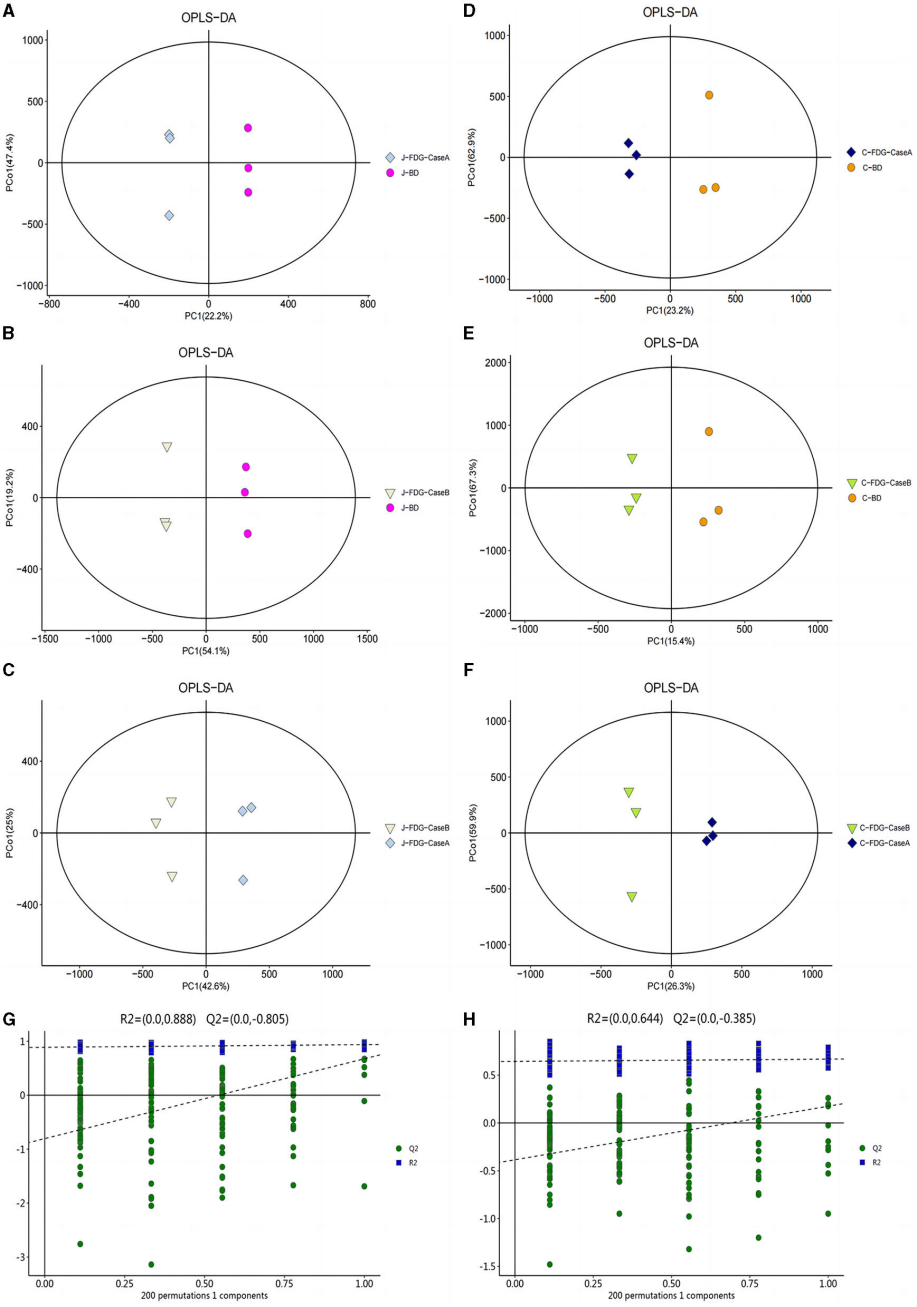
OPLS-DA for data distribution. 2D score scatter plot of the PCA model for the **(A)** J-FDG-Case A vs. BD (R^2^X = 0.786), **(B)** J-FDG-Case B vs. BD (R^2^X = 0.781), **(C)** J-FDG-Case B vs. J-FDG-Case A (R^2^X = 0.810), **(D)** C-FDG-Case A vs. BD (R^2^X = 0.828), **(E)** C-FDG-Case B vs. BD (R^2^X = 0.835), and **(F)** C-FDG-B vs. C-FDG-Case A comparisons, respectively (R^2^X = 0.818; *p* < 0.05). Permutation plots for the OPLS-DA model show R^2^ (blue) and Q^2^ (green) values. The results of the permutation test strongly indicate that the original model was valid [**(G, H)**, R^2^ intercept = 0.888/0.644, Q^2^ intercept = −0.805/−0.385]. BD, basal diet group; FDG-Case A and FDG-Case B: 15% FDG group and 30% FDG group.

An LC-MS full-scan detection method was used to illustrate the metabolic alterations in the jejunum and cecum tissues of Guanling crossbred cattle fed the FDG diets. In total, 8,464 features in ESI^+^ mode and 2,564 ion features in ESI^−^ mode were detectable, and 1,399 metabolites were identified. PCA scoring plots were initially generated using the processed data obtained from the metabolomics analysis of the jejunum and cecum to evaluate systematic errors in the samples and the trends within each comparison. As shown in Supplementary Figure S1, the quality control (QC) samples clustered in the center of the PCA score plots suggest that the analyses were robust and reproducible. Clear separation trends were observed in the FDG-Case A vs. BD, FDG-Case B vs. BD, and FDG-Case B vs. FDG-Case A comparisons, respectively (Figures 1A–F). To maximize the identification of differential metabolites between BD and FDG groups, OPLS-DA was used to observe the tendency for separation on the metabolic spectrum (Figures 2A–F). The validity of the model was evaluated through permutation testing (*n* = 200), with R^2^ values close to 1 and intercepts between the regression line and the vertical axis of Q^2^ <0. Moreover, Q^2^ gradually decreased with reduced displacement retention, affirming the absence of overfitting in the original model (Figures 2G, H). These results provide assurance for subsequent tests and analyses.

### 3.2. Differential metabolites in the intestinal tissues of FDG and BD groups

**Figure 3:**
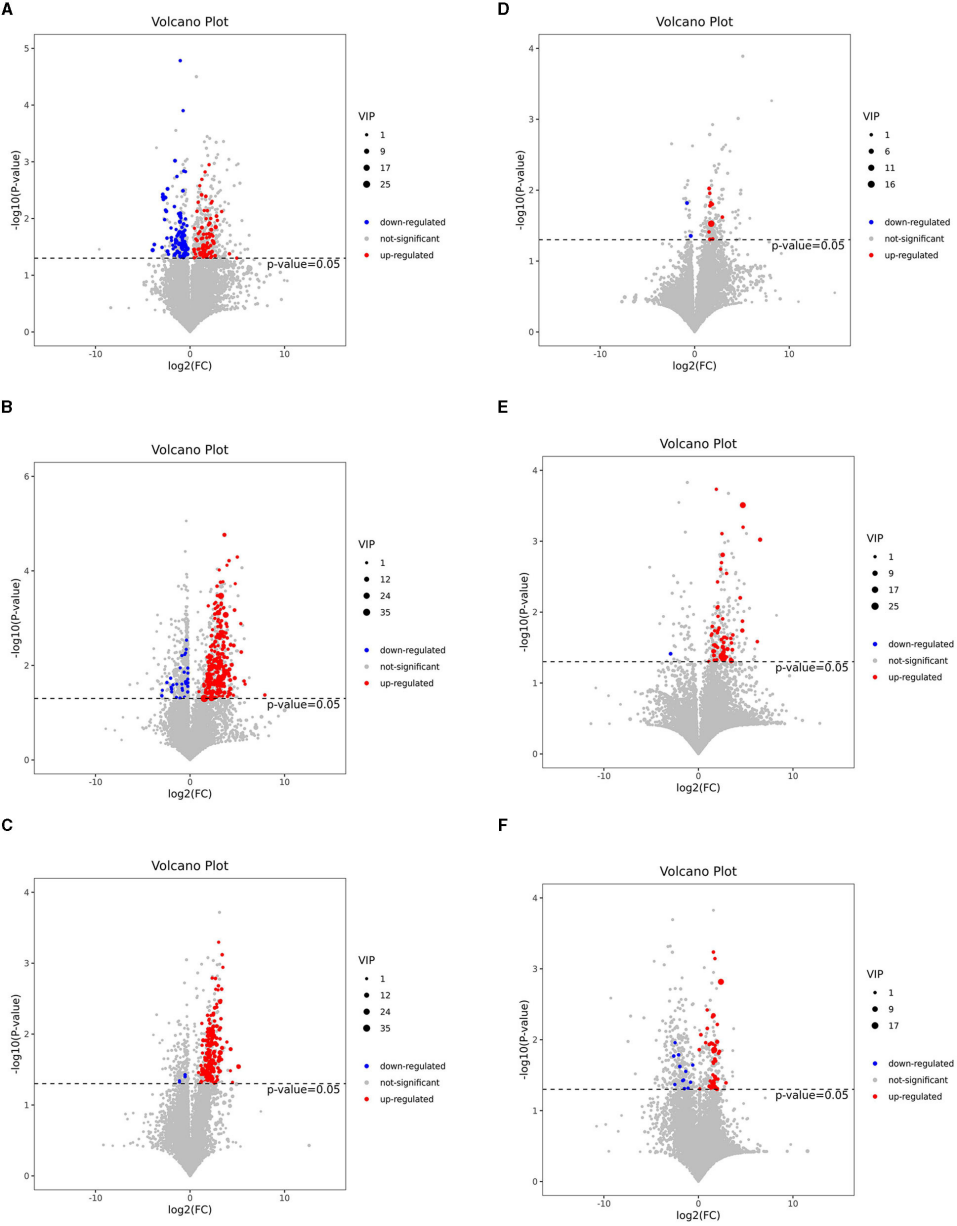
Volcano plots based on LC-MS of the jejunal and cecal metabolites. **(A–F)** Volcano plot based on LC-MS analyses of J-FDG-Case A vs. J-BD **(A)**, J-FDG-Case B vs. J-BD **(B)**, J-FDG-Case B vs. J-FDG-Case A **(C)**, C-FDG-Case A vs. C-BD **(D)**, C-FDG-Case B vs. C-BD **(E)**, and C-FDG-Case B vs. C-FDG-Case A **(F)** comparisons, respectively. The 1,399 significantly altered metabolites in the model group. Red and blue represent upregulated and downregulated metabolites, respectively, with VIP > 1, fold change> 1.3, and a *p* < 0.05. The gray area indicates unchanged metabolites with a fold change of <1.3 and a *p* > 0.05. BD, basal diet group; FDG-Case A and FDG-Case B: 15% FDG group and 30% FDG group.

To visualize the fold change (FC) analysis of 1,399 metabolites for quick identification of significantly differential metabolites, a volcano plot was created by transferring the FC values of each metabolite to log2(FC) and by transferring the *p-value* (*p* = 0.05) of Student's *t*-test to –log (*p*) while simultaneously meeting the variable importance in projection (VIP) values of > 1 for the first principal component. As shown in Figures 3A–F, a total of 178 metabolites [78 metabolites upregulated with log2(FC) > 0 and 100 metabolites downregulated with log2(FC) <0], 227 metabolites (121 upregulated and 106 downregulated), and 179 metabolites (174 upregulated and 5 downregulated) were identified in the J-FDG-Case A vs. J-BD, J-FDG-Case B vs. J-BD, and J-FDG-Case B vs. J-FDG-Case A comparisons, respectively, meeting the criteria of *p* < 0.05 and VIP value of >1. In addition, 12 metabolites (10 upregulated and 2 downregulated), 58 metabolites (57 upregulated and 1 downregulated), and 55 metabolites (43 upregulated and 12 downregulated) were identified in the C-FDG-Case A vs. C-BD, C-FDG-Case B vs. C-BD, and C-FDG-Case B vs. C-FDG-Case A comparisons, respectively.

**Figure 4:**
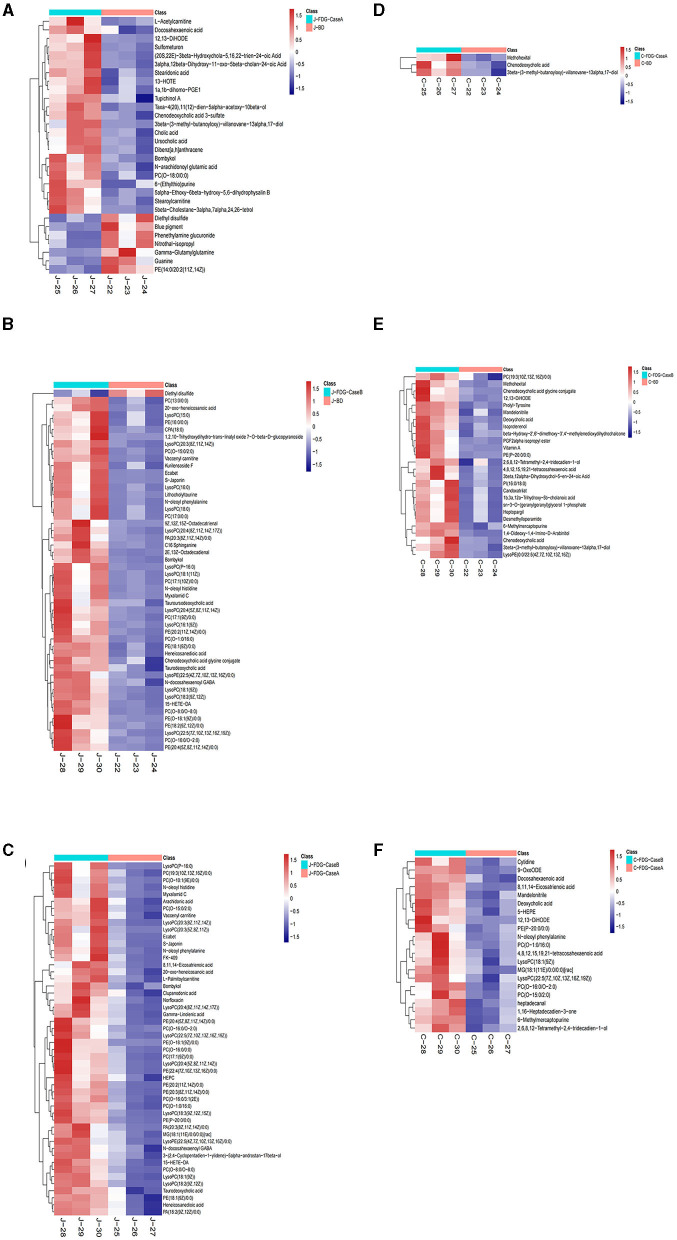
Heatmap of the top 50 differential metabolites in the jejunum and cecum. Heatmap plot based on LC-MS analyses of J-FDG-Case A vs. J-BD **(A)**, J-FDG-Case B vs. J-BD **(B)**, J-FDG-Case B vs. J-FDG-Case A **(C)**, C-FDG-Case A vs. C-BD **(D)**, C-FDG-Case B vs. C-BD **(E)**, and C-FDG-Case B vs. C-FDG-Case A **(F)** comparisons, respectively. The graph displays the differential metabolites on the Y-axis and the sample names on the X-axis. The color gradient from blue to red represents the expression abundance of the metabolites, with red indicating higher expression abundance and blue indicating lower expression abundance. BD, basal diet group; FDG-Case A and FDG-Case B: 15% FDG group and 30% FDG group.

The levels of the top 50 differential metabolites were visualized by a heatmap depicted in Figures 4A–F, in which colors represent increased (red) or decreased (blue) abundance, with the intensity reflecting the corresponding concentration. Of these, the relative richness (referring to the ratio of FDG/BD) of 23, 49, and 50 metabolites were decreased in the J-FDG-Case A vs. J-BD, J-FDG-Case B vs. J-BD, and J-FDG-Case B vs. J-FDG-Case A comparisons, respectively. Conversely, the richness of 7, 1, and 0 metabolites was significantly increased in the aforementioned jejunum comparisons, respectively. As for cecum samples, the relative richness of 3, 26, and 21 metabolites was decreased in the C-FDG-Case A vs. C-BD, C-FDG-Case B vs. C-BD, and C-FDG-Case B vs. C-FDG-Case A comparisons, respectively. However, no upregulated differential metabolites were found in the aforementioned cecum comparisons, respectively.

**Figure 5:**
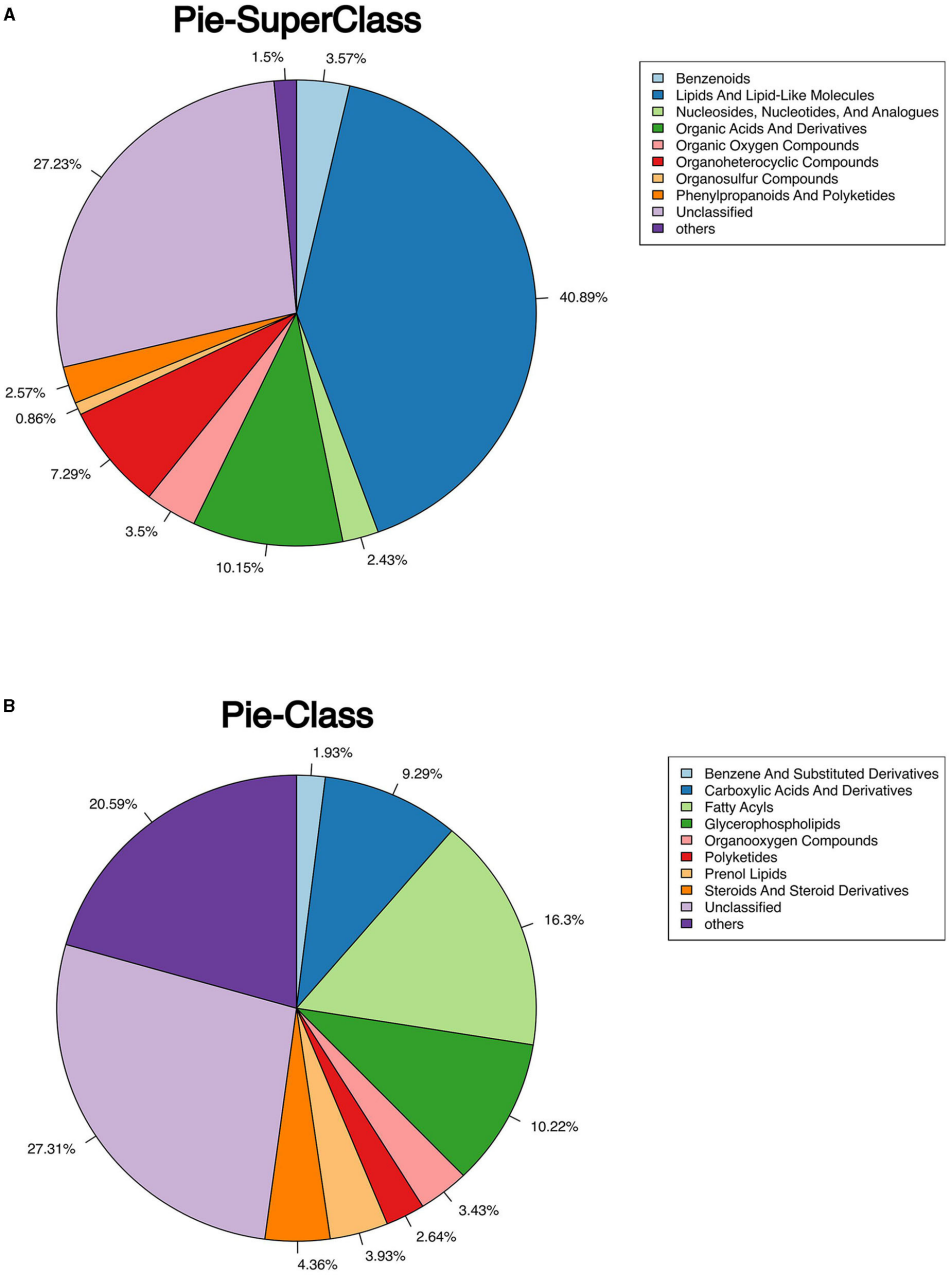
Pie chart shows the classification of total significant metabolites. **(A)** Classify total metabolites into superclasses. **(B)** The total metabolites are further divided into classes.

The classification of all significantly differential metabolites is shown in Figures 5A, B, and Tables 1, 2 offer detailed information on the top 20 differential metabolites. Of these, in the J-FDG-Case A vs. J-BD comparison, they were mainly classified into lipids and lipid-like molecules (fatty acyls, steroids and steroid derivatives, and prenol lipids) and organoheterocyclic compounds (imidazopyrimidines). In the J-FDG-Case B vs. J-BD comparison, the differential metabolites were mainly classified into lipids and lipid-like molecules (fatty acyls, steroids and steroid derivatives, and glycerophospholipids), organic acids and derivatives (carboxylic acids and derivatives), and organic oxygen compounds (organooxygen compounds). In the J-FDG-Case B vs. J-FDG-Case A comparison, the differential metabolites were mainly classified into lipids and lipid-like molecules (glycerophospholipids, fatty acyls, and prenol lipids), organic acids and derivatives (carboxylic acids and derivatives), and organoheterocyclic compounds (tetrahydroisoquinolines). As for cecum, in the C-FDG-Case A vs. C-BD comparison, the differential metabolites were mainly classified into lipids and lipid-like molecules (steroids and steroid derivatives, and prenol lipids) and organoheterocyclic compounds (diazines). In the C-FDG-Case B vs. C-BD, the differential metabolites were mainly classified into lipids and lipid-like molecules (fatty acyls, steroids and steroid derivatives, and glycerophospholipids), organoheterocyclic compounds (imidazopyrimidines and diazines), and benzenoids (phenols, benzene, and substituted derivatives). In C-FDG-Case B vs. C-FDG-Case A, the differential metabolites were mainly classified into lipids and lipid-like molecules (fatty acyls, glycerophospholipids, and steroids and steroid derivatives), organoheterocyclic compounds (imidazopyrimidines), and organic acids and derivatives (carboxylic acids and derivatives). Collectively, these results indicate that the metabolite profiles in the jejunum and cecum are predominantly affected by the 15% FDG and 30% FDG diets.

**Table 1:** Top 20 differential metabolites in the jejunum of experimental Guanling crossbred cattle determined by ultra-performance liquid chromatography-mass spectrometry (UPLC-MS)-based non-targeted metabonomics.

**Metabolites**	**Ion mode**	**VIP^a^**	***p* ^b^**	**log2(FC)^c^**
**J-FDG-Case A vs. J-BD**				
Diethyl disulfide	Neg	7.1244	0.0340	−0.2873
Bombykol	Pos	5.1151	0.0298	1.4149
L-Acetylcarnitine	Pos	3.5543	0.0434	1.1043
Stearoylcarnitine	Pos	3.5095	0.0122	2.0603
Tupichinol A	Neg	3.5076	0.0359	1.4598
Phenethylamine glucuronide	Pos	3.3073	0.0379	−2.3854
Cholic acid	Pos	3.2445	0.0157	2.2476
Ursocholic acid	Pos	3.0216	0.0255	2.4614
Taxa-4(20),11(12)-dien-5alpha-acetoxy-10beta-ol	Neg	2.4731	0.0038	1.2236
(20S,22E)-3beta-Hydroxychola-5,16,22-trien-24-oic Acid	Pos	2.3847	0.0091	2.8009
13-HOTE	Neg	2.1218	0.0297	1.4413
Blue pigment	Pos	1.9577	0.0250	−1.4026
5beta-Cholestane-3alpha,7alpha,24,26-tetrol	Pos	1.7963	0.0185	1.9972
3beta-(3-methyl-butanoyloxy)-villanovane-13alpha,17-diol	Neg	1.7175	0.0419	4.1676
5alpha-Ethoxy-6beta-hydroxy-5,6-dihydrophysalin B	Pos	1.6854	0.0222	1.3329
Nitrothal-isopropyl	Pos	1.5823	0.0323	−2.9587
Guanine	Pos	1.4658	0.0426	−2.2765
1a,1b-dihomo-PGE1	Neg	1.4361	0.0296	1.7224
N-arachidonoyl glutamic acid	Neg	1.4229	0.0100	2.1913
PE [14:0/20:2(11Z, 14Z)]	Pos	1.4122	0.0105	−2.6855
**J-FDG-Case B vs. J-BD**				
Tauroursodeoxycholic acid	Neg	27.7683	0.0488	2.2727
PE [18:1(9Z)/0:0]	Pos	15.1901	0.0008	2.9973
2E,13Z-Octadecadienal	Pos	14.2097	0.0023	3.2346
Chenodeoxycholic acid glycine conjugate	Neg	14.0927	0.0165	1.9299
LysoPC (16:0)	Neg	11.2534	0.0202	3.0580
N-docosahexaenoyl GABA	Pos	10.0703	0.0113	2.2070
LysoPC (15:0)	Neg	9.9910	0.0372	2.3170
LysoPC (18:0)	Neg	9.4316	0.0383	2.8572
LysoPC [18:1(11Z)]	Neg	9.1001	0.0162	3.5686
LysoPC [18:1(9Z)]	Pos	7.2734	0.0038	3.5445
Taurodeoxycholic acid	Pos	7.0707	0.0085	1.9652
PE [O-18:1(9Z)/0:0]	Pos	6.4751	0.0312	4.2610
Ecabet	Neg	6.2775	0.0149	4.0447
PC [17:1(10Z)/0:0]	Neg	6.1893	0.0174	3.5836
PC (17:0/0:0)	Neg	6.1601	0.0388	2.9031
CPA (18:0)	Pos	5.9387	0.0260	3.3299
LysoPC [18:2(9Z,12Z)]	Pos	5.5787	0.0020	3.3941
PC (13:0/0:0)	Pos	5.4871	0.0077	2.5790
PE [18:2(9Z,12Z)/0:0]	Neg	5.4821	0.0377	3.6312
Bombykol	Pos	5.4390	0.0025	2.8059
**J-FDG-Case B vs. J-FDG-Case A**				
PE [18:1(9Z)/0:0]	Pos	15.0548	0.0228	1.7003
N-docosahexaenoyl GABA	Pos	10.1249	0.0314	1.3234
Taurodeoxycholic acid	Pos	8.2977	0.0191	1.8920
PE [O-18:1(9Z)/0:0]	Pos	8.1763	0.0288	5.1397
LysoPC [18:1(9Z)]	Pos	7.9866	0.0136	2.3970
Ecabet	Neg	7.0896	0.0237	3.0430
LysoPC [18:2(9Z,12Z)]	Pos	6.5028	0.0035	3.1808
PC(O-15:0/2:0)	Pos	5.1363	0.0185	2.4912
PC(O-1:0/16:0)	Pos	5.1053	0.0078	2.4572
Bombykol	Pos	5.0985	0.0122	1.3910
S-Japonin	Neg	5.0836	0.0302	2.3950
PC(O-16:0/O-2:0)	Pos	4.7225	0.0336	2.5034
15-HETE-DA	Neg	4.5233	0.0106	1.8692
LysoPC [20:3(8Z,11Z,14Z)]	Pos	4.5041	0.0034	3.1663
LysoPC (P-16:0)	Pos	4.3641	0.0163	4.2970
PC (O-8:0/O-8:0)	Pos	4.3204	0.0077	2.8359
PE [20:4(5Z,8Z,11Z,14Z)/0:0]	Neg	4.2639	0.0368	1.9253
N-oleoyl phenylalanine	Pos	4.2077	0.0177	2.5725
N-oleoyl histidine	Neg	4.1058	0.0409	2.2735
Norfloxacin	Pos	3.6668	0.0472	1.5510

^a^VIP was obtained from the OPLS-DA model.

^b^p were calculated using t-test analysis, P <0.05.

^c^FC was calculated based on a binary logarithm for J-FDG-Case A vs. J-BD, J-FDG-Case B vs. J-BD, and J-FDG-Case B vs. J-FDG-Case A comparisons, respectively.

Data are presented as means M ± SD (n = 3).

**Table 2:** Top 20 differential metabolites in the cecum of experimental Guanling crossbred cattle determined by ultra-performance liquid chromatography-mass spectrometry (UPLC-MS)-based non-targeted metabonomics.

**Metabolites**	**Ion mode**	**VIP^a^**	***p* ^b^**	**log2(FC)^c^**
**C-FDG-Case A vs. C-BD**				
Chenodeoxycholic acid	Neg	13.1623	0.0297	1.7503
3beta-(3-methyl-butanoyloxy)-villanovane-13alpha,17-diol	Neg	1.9177	0.0095	1.5386
Methohexital	Pos	1.3455	0.0240	2.9305
**C-FDG-Case B vs. C-BD**				
1b,3a,12a-Trihydroxy-5b-cholanoic acid	Neg	27.0879	0.0420	2.5234
Chenodeoxycholic acid	Neg	21.1430	0.0451	2.6383
Chenodeoxycholic acid glycine conjugate	Neg	8.2490	0.0486	3.4802
6-Methylmercaptopurine	Neg	5.1395	0.0016	2.5675
4,8,12,15,19,21-tetracosahexaenoic acid	Pos	5.0808	0.0372	2.6411
Heptopargil	Neg	4.2787	0.0310	2.5795
PGF2alpha isopropyl ester	Pos	4.1446	0.0182	4.6435
3beta,12alpha-Dihydroxychol-5-en-24-oic Acid	Pos	3.7693	0.0332	3.0863
3beta-(3-methyl-butanoyloxy)-villanovane-13alpha,17-diol	Neg	2.6737	0.0086	2.0037
Beta-Hydroxy-2′,6′-dimethoxy-3′,4′-methylenedioxydihydrochalcone	Pos	2.4738	0.0356	1.5870
PI (16:0/18:0)	Neg	2.4363	0.0340	3.5853
Deoxycholic acid	Pos	2.3023	0.0063	4.4238
12,13-DiHODE	Neg	2.1573	0.0244	2.4303
Desmethylloperamide	Neg	2.1459	0.0269	2.6648
sn-3-O-(geranylgeranyl) glycerol 1-phosphate	Neg	2.0412	0.0228	2.6877
Methohexital	Pos	1.9445	0.0210	3.6401
Isoproterenol	Pos	1.7951	0.0159	1.4726
Vitamin A	Pos	1.6360	0.0261	6.2312
2,6,8,12-Tetramethyl-2,4-tridecadien-1-ol	Neg	1.3780	0.0499	1.0910
Prolyl-Tyrosine	Pos	1.3770	0.0008	2.4883
**C-FDG-Case B vs. C-FDG-Case A**				
4,8,12,15,19,21-tetracosahexaenoic acid	Pos	4.5599	0.0493	1.9536
6-Methylmercaptopurine	Neg	4.5558	0.0045	1.5896
N-oleoyl phenylalanine	Pos	2.8343	0.0462	1.7273
LysoPC [18:1(9Z)]	Pos	2.7302	0.0455	1.1595
Heptadecanal	Neg	2.1881	0.0116	1.1587
Deoxycholic acid	Pos	1.9949	0.0146	2.2289
PC (O-16:0/O-2:0)	Pos	1.9754	0.0207	1.5674
PC (O-15:0/2:0)	Pos	1.9356	0.0385	1.6032
12,13-DiHODE	Neg	1.9150	0.0360	1.7920
5-HEPE	Pos	1.8851	0.0112	1.3511
MG [18:1(11E)/0:0/0:0] [rac]	Pos	1.8326	0.0188	1.7423
Cytidine	Pos	1.7980	0.0115	1.5105
9-OxoODE	Pos	1.6395	5.0593	1.3115
1,16-Heptadecadien-3-one	Neg	1.6349	0.0069	0.9395
PC (O-1:0/16:0)	Pos	1.6295	0.0487	1.3687
2,6,8,12-Tetramethyl-2,4-tridecadien-1-ol	Neg	1.2665	0.0110	0.7710
Mandelonitrile	Pos	1.2496	0.0061	1.9975
LysoPC [22:5(7Z,10Z,13Z,16Z,19Z)]	Pos	1.1160	0.0382	1.2460
PE (P-20:0/0:0)	Pos	1.1051	0.0458	1.7718
Docosahexaenoic acid	Pos	1.0425	0.0038	0.9268

^a^VIP was obtained from the OPLS-DA model.

^b^p were calculated using t-test analysis, a p-value of <0.05.

^c^FC was calculated based on a binary logarithm C-FDG-Case A vs. C-BD, C-FDG-Case B vs. C-BD, and C-FDG-Case B vs. C-FDG-Case A comparisons, respectively.

Data are presented as means M ± SD (n = 3).

### 3.3. Metabolic pathway analysis

**Figure 6:**
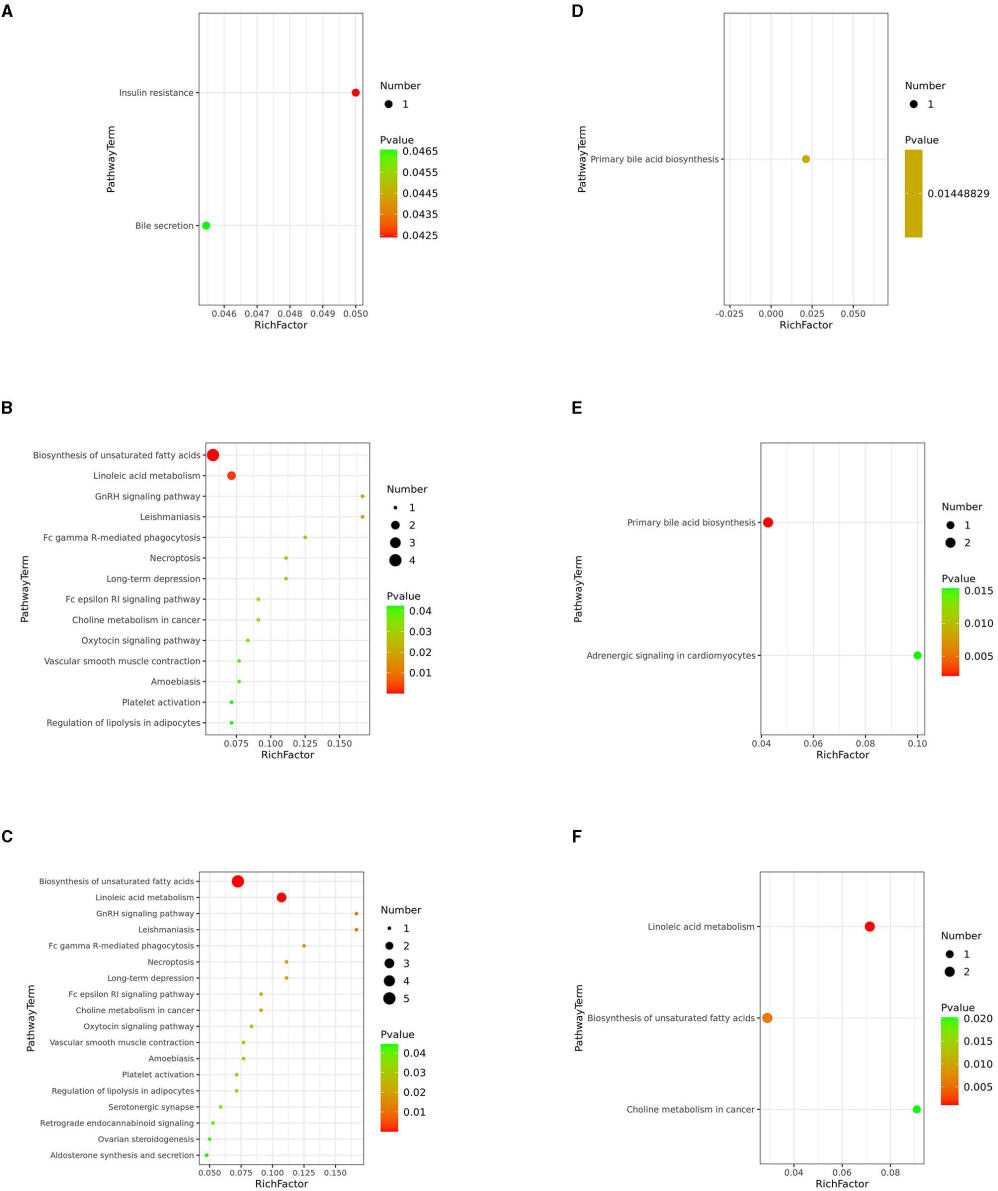
KEGG pathway analysis. Differentially altered metabolic pathways enriched in the J-FDG-Case A vs. J-BD **(A)**, J-FDG-Case B vs. J-BD **(B)**, J-FDG-Case B vs. J-FDG-Case A **(C)**, C-FDG-Case A vs. C-BD **(D)**, C-FDG-Case B vs. C-BD **(E)**, and C-FDG-Case B vs. C-FDG-Case A comparisons **(F)**, respectively. The graph displays the metabolic pathways on the Y-axis and the enrichment factor (Rich factor = The number of significantly different metabolites/The total number of metabolites in the pathway) on the X-axis. A higher Rich factor indicates a higher degree of enrichment, calculated by the number of significantly different metabolites divided by the total number of metabolites in the pathway. The color gradient from green to red represents decreasing *p*-values. Larger dots indicate more metabolites enriched in the pathway. BD, basal diet group; FDG-Case A and FDG-Case B: 15% FDG group and 30% FDG group.

Next, we investigated potential metabolic pathways responsible for the observed changes in the intestinal metabolic profile associated with FDG diets. The identified metabolites were annotated with KEGG and HMDB. Results showed that a total of 40 metabolic pathways were identified (*p* < 0.05). Among them, there were 2 (insulin resistance and bile secretion), 14 (including biosynthesis of unsaturated fatty acids and terpenoid backbone biosynthesis), and 18 (including biosynthesis of unsaturated fatty acids, linoleic acid metabolism, and GnRH signaling pathway) significant metabolic pathways (*p* < 0.05) enriched in the J-FDG-Case A vs. J-BD, J-FDG-Case B vs. J-BD, and J-FDG-Case B vs. J-FDG-Case A comparisons, respectively, and 1 (primary bile acid biosynthesis), 2 (primary bile acid biosynthesis and adrenergic signaling in cardiomyocytes), and 3 (linoleic acid metabolism, biosynthesis of unsaturated fatty acids, and choline metabolism in cancer) significantly enriched metabolic pathways (*p* < 0.05) were obtained in the C-FDG-Case A vs. C-BD, C-FDG-Case B vs. C-BD, and C-FDG-Case B vs. C-FDG-Case A comparisons, respectively (Figures 6A–F). Furthermore, four significant pathways, including biosynthesis of unsaturated fatty acids, linoleic acid metabolism, insulin resistance, and primary bile acid biosynthesis [*p* < 0.05, –log(*p*)>1.3] were significantly enriched in Guanling crossbred cattle fed with FDG diets (Table 3). Among them, the insulin resistance pathway was found to be specific to the jejunum, while the primary bile acid biosynthesis pathway was specific to the cecum. The biosynthesis of unsaturated fatty acids and linoleic acid metabolism pathways were shared between the jejunum and cecum.

**Table 3:** List of metabolic pathways with significant difference in the FDG group compared to the BD group.

**Annotation**	**In set**	**-log(*p*)**	**FDR**	***p***
Insulin resistance	1	1.3725	0.1406	0.0424
Biosynthesis of unsaturated fatty acids	6	7.1163	0.0000	0.0011
Linoleic acid metabolism	4	4.7053	0.0002	0.0063
Primary bile acid biosynthesis	2	2.6992	0.0079	0.0144

Based on the selection threshold of –log(p) > 1.3 and a p <0.05, four significant target pathways were obtained. In set means the number of differential metabolites involved in this pathway; the p-value is calculated from the enrichment analysis; FDR value is the false discovery rate to adjust the p-values.

## 4. Discussion

Guizhou province of China is well-known for the production of Jiang-flavor Moutai liquor, and a large number of distillers' grain (DG) resources are generated as byproducts with an annual output exceeding 19 million tons (23). DGs are nutrition-rich byproducts and are abundant in various crude substances (24, 25). Evidence has revealed that incorporating fermented distillers' grains (FDGs) as a feed additive is beneficial for the growth and development of ruminants, such as cattle and sheep. This is because fermentation treatment of DG increases feed palatability and intramuscular protein and fat content and stimulates rumen fermentation and intestinal development in animals (9, 26, 27). Additionally, the fermentation of DG improves its nutrients, thus promoting their utilization in feed production. Alterations in animal intestinal flora structure have been identified in several FDG diet-related studies (28–31). However, few studies have focused on the metabolic alterations of animal intestinal tissues to date. The present study showed that the intestinal metabolomic patterns of Guanling crossbred cattle fed the FDG diets were significantly different from those of BD diet-feeding cattle. FDG diets exert growth-promoting and anti-inflammatory potential through the regulation of fatty acyls, steroids, and steroid derivatives. The levels of several metabolites, including l-acetylcarnitine, 8,11,14-eicosatrienoic acid, adrenic acid, arachidonic acid, clupanodonic acid, gamma-Linolenic acid, docosahexaenoic acid, 9-OxoODE, chenodeoxycholic acid, and chenodeoxycholic acid glycine conjugate, were notably increased in the FDG group. Based on the identified differential metabolites, insulin resistance, biosynthesis of unsaturated fatty acids, linoleic acid metabolism, and primary bile acid biosynthesis metabolic pathways were significantly enriched in the FDG group, where biosynthesis of unsaturated fatty acids and linoleic acid metabolism are shared by the jejunum and cecum samples, suggesting potentially specific functions in the improvement of intestinal inflammation in cattle.

Insulin resistance is a specific metabolic pathway significantly enriched in the jejunum of the FDG-Case A group. The J-FDG-Case A group exhibits substantial upregulation of L-acetylcarnitine, which is an amino acid derivative capable of regulating insulin resistance and impacting gut health (Figure 6A). As a key site for food digestion and nutrient absorption, the intestinal tract also plays a vital role in influencing insulin sensitivity and metabolic function and can simultaneously be regulated by insulin (32). L-acetylcarnitine is an intermediate metabolite in insulin resistance, which increases fatty acid oxidation during lipid metabolism and promotes fatty acid utilization when the body's response to insulin is reduced. This results in elevated blood glucose and insulin overproduction utilization and reduced fat deposition and adipose tissue inflammation, thus improving insulin resistance and reducing oxidative stress and inflammatory responses caused by insulin resistance, suggesting the antioxidant and anti-inflammatory properties of L-acetylcarnitine (33). Our study suggests that FDG diets may help maintain intestinal epithelial cell integrity and function and sustain intestinal health by improving insulin resistance through L-acetylcarnitine, thereby reducing oxidative stress and promoting the growth and development of Guanling crossbred cattle. The complex interplay between insulin resistance and intestinal metabolism entails the regulation of multiple factors, and the underlying mechanisms remain to be further studied to gain new insights into the onset and progression of insulin resistance and devise new intervention strategies to improve insulin sensitivity and reduce the risk of metabolic disorders.

Primary bile acid biosynthesis is a metabolic pathway specific to the cecum, wherein chenodeoxycholic acid (CDCA) and chenodeoxycholic acid glycine conjugate (CDCA-Gly) are the main metabolites. The levels of CDCA and CDCA-Gly were significantly higher in both the C-FDG-Case A vs. C-BD and the C-FDG-Case B vs. C-BD comparisons (Figures 6D, E). CDCA and CDCA-Gly are produced through primary bile acid biosynthesis and play a critical role in the absorption and digestion of fat in the gallbladder and small intestine (34). Higher concentrations of CDCA and CDCA-Gly have been evidenced to promote gallbladder contraction, enhance the formation of bile and fatty acids to form an emulsion in the intestinal flow, which in turn improves the intestinal absorption of fatty acids, glycerol, and cholesterol, and influence the growth and composition of intestinal microorganisms, which ultimately promotes intestinal digestion and absorption (35, 36). The present study suggests that FDG diets may increase the concentrations of CDCA and CDCA-Gly, thereby stimulating the intestinal bile circulation system to reduce bile acid synthesis. Such promotion of intestinal digestion and absorption is beneficial for the intestinal metabolism of Guanling crossbred cattle. However, it is important to note that over-regulation may adversely affect the synthesis and circulation of bile acids. Therefore, it is necessary to control the metabolic concentrations of CDCA and CDCA-Gly.

The biosynthesis of unsaturated fatty acids and linoleic acid metabolism pathways are shared by the jejunum and cecum, and both of them belong to fatty acid metabolism. 8,11,14-Eicosatrienoic acid, adrenic acid, arachidonic acid, clupanodonic acid, gamma-linolenic acid, docosahexaenoic acid, gamma-linolenic acid, and 9-OxoODE are the initial substrates for the biosynthesis of unsaturated fatty acids and linoleic acid metabolism. These metabolites were significantly upregulated in J-FDG-Case B vs. J-BD, J-FDG-Case B vs. J-FDG-Case A, and C-FDG-Case B vs. C-FDG-Case A comparisons, respectively. In the biosynthesis of unsaturated fatty acids, 8,11,14-eicosatrienoic acid, clupanodonic acid, and docosahexaenoic acid (DHA) are precursors of ω-3 series fatty acids, which are involved in the synthesis of ω-3 polyunsaturated fatty acids and the regulation of nerve and brain development, immune function, and inflammatory response (37). Arachidonic acid (AA), gamma-linolenic acid, and adrenic acid are the precursors of ω-6 fatty acids and are involved in the synthesis of ω-6 polyunsaturated fatty acids, which are involved in the synthesis of cell membranes, cell signaling, and important physiologically active substances such as prostaglandins and leukotrienes possessing anti-inflammatory and immunomodulatory effects (38–43). Overall, these metabolites work together in the biosynthesis process to create a variety of unsaturated fatty acids that play important physiological functions and regulatory roles. During linoleic acid metabolism, 8,11,14-eicosatrienoic acid acts as a precursor of AA and forms interrupted aromatic terminal ketone (EETA) catalyzed by cytochrome P450 (44). 9-OxoODE is formed by cytochrome P450 enzymes and cyclooxygenases, which can inhibit EET hydrolases, leading to an increased concentration of EETs. This has an impact on the angiotensin pathway, inflammatory processes, and apoptosis. AA and γ-linolenic acid are important metabolites of linoleic acid, both of which are converted into prostaglandins (PGs) and leukotrienes through the activity of cyclo-oxidases and lipoxygenases, respectively (45–47). The aforementioned studies demonstrate that γ-linolenic acid, AA, and DHA possess anti-inflammatory properties. γ-linolenic acid and DHA are the principal fatty acids present in brain and retinal tissues, which can promote the differentiation function of immunomodulatory cells, enhance immune response, maintain the integrity of the intestinal mucosal barrier, and reduce mucosal oxidative damage to promote intestinal health. This study suggests that feeding FDG diets may stimulate the biosynthesis of unsaturated fatty acids and linoleic acid metabolism in Guanling crossbred cattle, resulting in elevated levels of these metabolites. These may have anti-inflammatory, immunomodulatory, and intestinal health-promoting effects, thereby benefiting the growth and development of Guanling crossbred cattle. However, it is important to note that the specific effects of the FDG diets may vary according to individual differences, background health status, and other environmental factors. Therefore, further studies are still needed to investigate the effects and potential mechanisms of action of these metabolites on the gut health of animals.

## 5. Conclusion

In conclusion, our results suggest that the FDG diet may promote the gut health and immunity of Guanling crossbred cattle by modulating the metabolic patterns of lipid compounds and lipid-related metabolic pathways, such as biosynthesis of unsaturated fatty acids and linoleic acid metabolism, and primary bile acid biosynthesis. However, this study is merely a pilot study, and further in-depth analysis with a large number of animals is needed to confirm the specific regulatory functions and their potential mechanisms of action. Our findings contribute to further understanding of the nutritive and regulatory functions of the FDG diet for Guanling crossbred cattle and their underlying mechanisms as well as provide some references for FDG applications as a feed resource.

## Data availability statement

The original contributions presented in the study are included in the article/Supplementary material, further inquiries can be directed to the corresponding author/s.

## Ethics statement

The animal studies were approved by EAE-GZu-2020-E018, 2 September 2020. The studies were conducted in accordance with the local legislation and institutional requirements. Written informed consent was obtained from the owners for the participation of their animals in this study.

## Author contributions

XL, EZ, ZCheng, and CC conceived the study. XL, TZ, DX, and MZ performed the experiments. XL, JZ, RZ, GH, EZ, ZCheng, and CC analyzed experimental results and data. GH, BZ, KW, CC, and SM assisted with animal experiments. XL wrote the manuscript. All authors have read and agreed to the published version of the manuscript.
