# Supplementary material for: The effect of feeding fermented distillers' grains diet on the intestinal metabolic profile of Guanling crossbred cattle
Source: Front Vet Sci. 2023 Oct 20;10:1238064. doi: 10.3389/fvets.2023.1238064 (PMC10622970; doi:10.3389/fvets.2023.1238064)
Supplement: Supplementary file 1 [file Data_Sheet_1.doc]

Supplementary Figures:


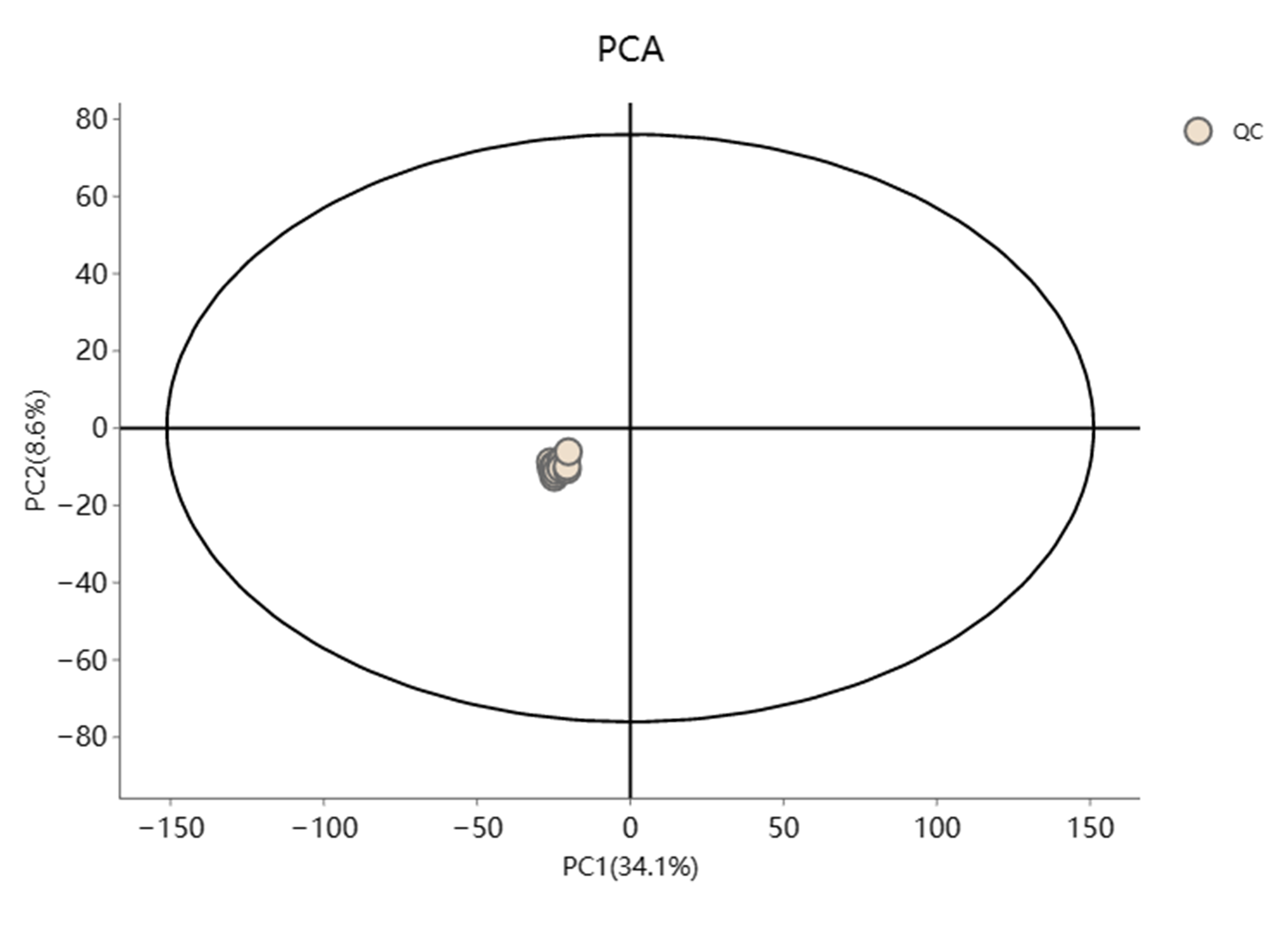


Fig.1 PCA for data distribution. 2D score scatter plot of the PCA model for the QC samples.

Supplementary Tables:

**Table S1** Composition and nutritional content of experimental diets varying in

levels of fermented distillers’ grain (FDG)

| Items | BD | R1 | R2 | FDG |
| --- | --- | --- | --- | --- |
| Ingredient, % DM inclusion | | | | |
| Pennisetum Sinese Roxb | 60 | 60 | 60 |  |
| Groundcorn | 20 | 10 | 0 |  |
| Soybean meal | 6 | 3 | 0 |  |
| Rapeseed meal | 4 | 2 | 0 |  |
| Wheat bran | 8 | 8 | 8 |  |
| FDG | 0 | 15 | 30 |  |
| Supplement | | | | |
| Rock fine | 1.4 | 1.4 | 1.4 |  |
| Salt | 0.05 | 0.05 | 0.05 |  |
| Calcium bisulfate | 0.42 | 0.42 | 0.42 |  |
| Sodium sulfate | 0.08 | 0.08 | 0.08 |  |
| Microelement additive | 0.05 | 0.05 | 0.05 |  |
| Total 100 | 100 | 100 | 100 |  |
| Chemical analysis | | | | |
| Dry matter (%) | 70.58 7 | 70.68 | 71.20 | 31.50 |
| Gross energy (MJ/kg DM) | 15.76 | 16.60 | 16.70 | 18.69 |
| Crude protein (% DM) | 11.40 | 12.64 | 13.88 | 21.88 |
| Neutral detergent fiber (% DM) | 54.47 | 55.47 | 56.47 | 36.56 |
| Acid detergent fiber (% DM) | 29.37 | 32.96 | 36.54 | 23.33 |
| Ether extract (% DM) | 3.30 | 3.67 | 4.03 | 6.52 |
| Total P | 0.72 | 0.74 | 0.77 | 0.76 |
| Total K (% DM) | 1.22 | 1.15 | 0.95 | 0.45 |
| Calcium (% DM) | 0.82 | 0.83 | 0.83 | 0.43 |
| Amino acid (% DM) | | | | |
| Aspartic acid | 0.45 | 0.56 | 0.75 | 2.87 |
| Threonine | 0.22 | 0.30 | 0.40 | 1.39 |
| Serine | 0.25 | 0.31 | 0.46 | 1.89 |
| Glutamic acid | 0.85 | 1.26 | 2.13 | 4.23 |
| Proline | 0.24 | 0.38 | 0.63 | 3.49 |
| Glycine | 0.26 | 0.34 | 0.47 | 1.44 |
| Alanine | 0.35 | 0.55 | 0.87 | 2.26 |
| Valine | 0.26 | 0.36 | 0.52 | 1.62 |
| Methionine | 0.10 | 0.13 | 0.19 | 0.85 |
| Isoleucine | 0.21 | 0.30 | 0.42 | 1.43 |
| Leucine | 0.42 | 0.63 | 0.96 | 3.94 |
| Tyrosine | 0.23 | 0.29 | 0.36 | 1.53 |
| Phenylalanine | 0.34 | 0.43 | 0.56 | 1.93 |
| Lysine | 0.21 | 0.26 | 0.31 | 1.02 |
| Histidine | 0.35 | 0.42 | 0.50 | 0.92 |
| Arginine | 0.18 | 0.22 | 0.31 | 1.55 |

FDG, fermented distillers’ grain; BD, 0% FDG; R1, 15% FDG; R2, 30%

FDG; DM, dry matte.

**Table S2** Chromatographic gradient Conditions for UPLC-Q-TOF/MS analysis

| Time (min) | Flow rate ((mL /min) | Mobile phase A (%) | Mobile phase B (%) |
| --- | --- | --- | --- |
| 0 | 0.4 | 99 | 1 |
| 1 | 0.4 | 70 | 30 |
| 2.5 | 0.4 | 40 | 60 |
| 6.5 | 0.4 | 10 | 90 |
| 8.5 | 0.4 | 0 | 100 |
| 10.7 | 0.4 | 0 | 100 |
| 10.8 | 0.4 | 99 | 1 |
| 13 | 0.4 | 99 | 1 |

**Table S3** Chromatographic gradient Conditions for UPLC-Q-TOF/MS analysis

| MS conditions | |
| --- | --- |
| Capillary voltages | 2.5 kV（ES+）2.5 kV（ES-） |
| Declustering potential | 40 V  4 eV  115 ℃  450 m  900 L/h  50-1000 amu  0.2 s  0.02 s |
| Collision energy |
| Source temperature |
| Desolvation temperature |
| Desolvation gas flow |
| Mass range |
| Scan time |
| interscan delay |
